# Effective Treatment of a Malignant Breast Phyllodes Tumor with Doxorubicin-Ifosfamide Therapy

**DOI:** 10.1155/2019/2759650

**Published:** 2019-06-18

**Authors:** Shinya Yamamoto, Shigeru Yamagishi, Toshiro Kohno, Ryosuke Tajiri, Toshikazu Gondo, Noboru Yoshimoto, Nobuko Kusano

**Affiliations:** ^1^Department of Breast Surgery, Fujisawa City Hospital, 2-6-1 Fujisawa, Fujisawa City, Kanagawa 251-8550, Japan; ^2^Shonan Fujisawa Clinic, 1-15 Hanazawa-cho, Kugenuma, Fujisawa City, Kanagawa 251-0023, Japan; ^3^Department of Diagnostic Pathology, Fujisawa City Hospital, 2-6-1 Fujisawa, Fujisawa City, Kanagawa 251-8550, Japan; ^4^Department of Thoracic Surgery, Fujisawa City Hospital, 2-6-1 Fujisawa, Fujisawa City, Kanagawa 251-8550, Japan; ^5^Ambulatory Treatment Center, Fujisawa City Hospital, 2-6-1 Fujisawa, Fujisawa City, Kanagawa 251-8550, Japan

## Abstract

Malignant phyllodes tumors of the breast occur infrequently and are difficult to treat with chemotherapy. Here, we present an effective chemotherapy strategy for recurrent malignant breast phyllodes tumors. A 48-year-old woman was diagnosed with a malignant phyllodes tumor in her right breast and underwent total right mastectomy. One year later, the tumor recurred in the right (a 2.2 cm mass) and left (a 10 cm mass) lungs; pleural effusion was also observed in the left lung. Eight courses of doxorubicin-ifosfamide (AI) therapy were administered. After treatment, the right lung mass and pleural effusion regressed completely and the left lung mass regressed to 2 cm. In conclusion, AI therapy is useful for treating recurrent malignant breast phyllodes tumors.

## 1. Introduction

Malignant phyllodes tumors of the breast are infrequent, and effective chemotherapy strategies are lacking. Here, we describe an effective treatment strategy for recurrent malignant breast phyllodes tumors.

## 2. Case Presentation

**Figure 1:**
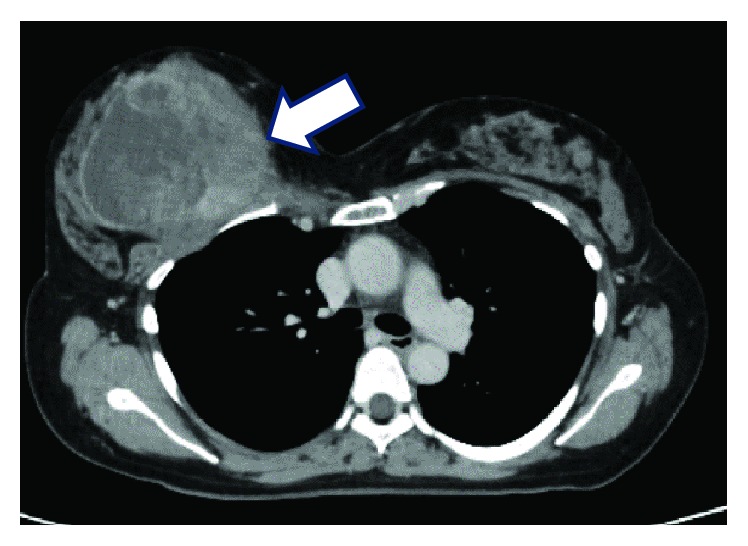
Computed tomography scan showing a 9-cm mass in the right breast (arrow).

A 48-year-old woman presented to our hospital with a mass in her right breast (Figure 1). A malignant phyllodes tumor was diagnosed via core needle biopsy, and right total mastectomy was performed. The pathological findings were consistent with the preoperative diagnosis, and the margins of the resected tissue were negative.

**Figure 2:**
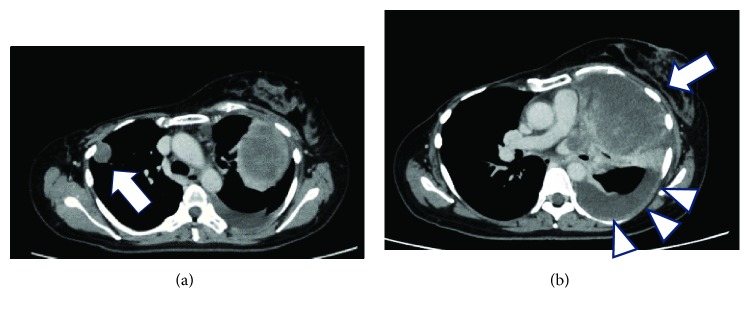
Computed tomography scan acquired before doxorubicin-ifosfamide (AI) chemotherapy showing (a) a 2.2 cm mass in the right lung (arrow) and (b) a 10 cm mass (arrow) and pleural effusion in the left lung (arrowheads).

One year later, the patient presented with cough and dyspnea. Computed tomography revealed a 2.2 cm mass in the right lung (Figure 2(a)) and 10 cm mass and pleural effusion in the left lung (Figure 2(b)). The masses were diagnosed as recurrent malignant phyllodes tumors. They were deemed unresectable because they were present in both lungs, and pleural dissemination was suspected.

**Figure 3:**
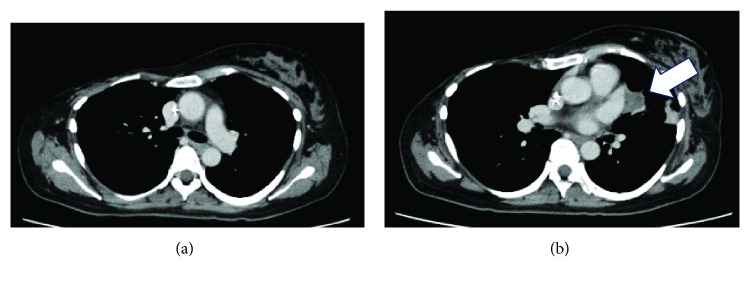
Computed tomography scan acquired after doxorubicin-ifosfamide (AI) chemotherapy showing (a) complete regression of the right lung mass and (b) partial regression of the left lung mass to 2 cm (arrow) and an absence of pleural effusion.

As an alternative treatment, we administered 8 courses of doxorubicin-ifosfamide (AI) therapy (30 mg/m^2^ doxorubicin on days 1-2 and 2 g/m^2^ ifosfamide on days 1-5) (Table 1). After chemotherapy, the right lung mass regressed completely (Figure 3(a)), the left lung mass regressed to 2 cm (Figure 3(b)), and pleural effusion was no longer detected. All 8 courses of AI therapy included mesna (sodium 2-mercaptoethane sulfonate) and sufficient infusion volumes to prevent ifosfamide-related hemorrhagic cystitis. Hemorrhagic cystitis did not occur during any of the courses.

Grade 4 neutropenia (as defined by the Common Terminology Criteria for Adverse Events (CTCAE)) occurred on day 15 of the first treatment cycle. To prevent the neutropenia from advancing, filgrastim, a granulocyte colony-stimulating factor, was administered on days 15 and 16 of the first cycle and pegfilgrastim, a persistent granulocyte colony-stimulating factor, was administered on day 8 or 9 of the following cycle. Febrile neutropenia was not observed during any of the courses.

We administered a diuretic drug at a concentration appropriate for the patient's weight when indicated, as well as a selective neurokinin 1 (NK1) receptor antagonist or other antiemetic. NK1 receptor antagonist administration was extended to 5 days owing to grade 2 nausea (as defined by the CTCAE) after the second cycle, and good control of the nausea was achieved. No other severe adverse events were noted. To avoid cardiotoxicity, AI therapy was terminated after 8 courses. Surgery was considered at this point but was refused by the patient. Thus, 4 courses of docetaxel (75 mg/m^2^) were administered instead.

**Figure 4:**
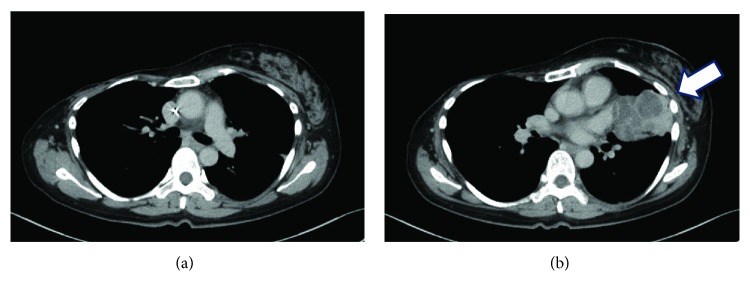
Computed tomography scan acquired after docetaxel chemotherapy showing (a) a lack of reappearance of the right lung mass and (b) an increase in the size of the left lung mass to 5.5 cm (arrow).

**Figure 5:**
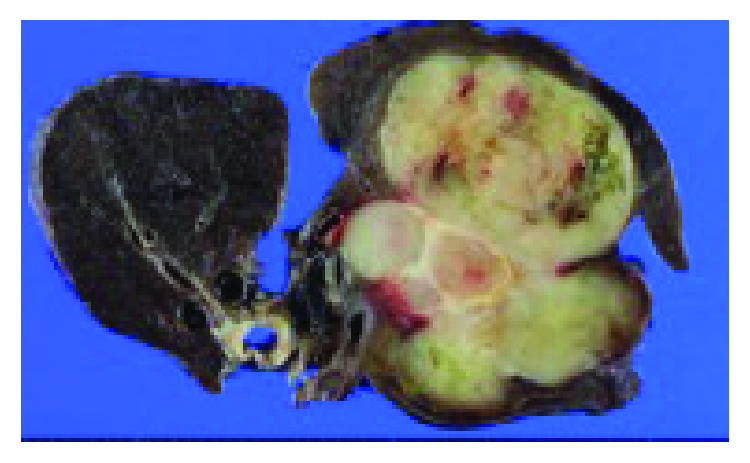
Pathological examination after lung surgery showing a 9 cm mass in the left lung reaching just under and partially adjacent to the pleura. There was no direct infiltration of the upper or the lower pulmonary vein or the pericardium.

**Figure 6:**
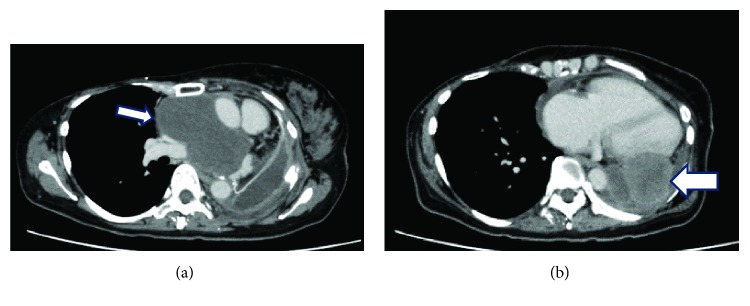
Computed tomography scan acquired 4 months after lung surgery showing (a) a 10 cm mass in the mediastinum (arrow) and (b) a 6 cm mass in the left thoracic cavity (arrow).

Although the right lung mass did not reappear (Figure 4(a)), the left lung mass increased in size to 5.5 cm (Figure 4(b)). As a result, the patient consented to surgery. Total left lung extraction and partial pericardial resection were performed. Pathological findings were consistent with a diagnosis of lung-metastatic breast phyllodes tumor, and curative resection was achieved (Figure 5). Four months after lung surgery, a 10 cm mass in the mediastinum (Figure 6(a)) and 6 cm mass in the left thoracic cavity (Figure 6(b)) were observed. The patient was therefore diagnosed with recurrent malignant phyllodes tumor of the left lung. One course of ifosfamide monotherapy (2 g/m^2^ on days 1-4) was administered. However, the patient's condition worsened abruptly before discharge and she died.

## 3. Discussion

Breast phyllodes tumors account for only 0.3%-0.9% of all breast tumors [1]. Based on their histological features, they are classified as benign, boundary, or malignant; 13%-40% of malignant tumors show metachronous distant metastases. Owing to the low frequency of distant metastasis, only a small number of retrospectively analyzed cases have been reported and a treatment strategy for malignant breast phyllodes tumors has not been established. Chemotherapy for unresectable distant metastases of malignant breast phyllodes tumors is generally similar to that used for soft tissue sarcomas [2]. The usefulness of anthracycline- and ifosfamide-based regimens [3, 4], as well as of high-capacity ifosfamide or anthracyclines plus granulocyte-macrophage colony-stimulating factor, for treatment of soft tissue sarcomas has been reported [5–7]. In accordance with previous reports [3–7], we administered AI therapy, using 60 mg/m^2^ doxorubicin and 10 g/m^2^ ifosfamide in each course. Because doxorubicin can be cardiotoxic, its total dose did not exceed 500 mg/m^2^. Regarding the dose indication of Adriamycin in our country, it is known that up to 500 mg is safe to administer.

Leyvraz et al. administered a combination of high-dose ifosfamide and high-dose doxorubicin to patients with soft tissue sarcomas [8], although at different doses than those used in our study. A total of 187 chemotherapy cycles were administered, and salvage therapy was also performed. The adverse events observed (and the percentage of cycles in which they occurred) were as follows: grade 3 neutropenia (59%), febrile episodes (29%), grade 3 thrombocytopenia (39%), anemia (27%), and transient microscopic hematuria (9%). One patient died of septic shock during the fourth cycle. There were no instances of severe renal toxicity.

In our study, the right lung mass completely regressed after 8 courses of AI therapy, and the left lung mass regressed considerably. Hence, AI therapy was very effective. After AI therapy, we considered surgery (which the patient refused) and ifosfamide single-agent therapy (which we ultimately rejected owing to slow tumor regression during the second half of AI therapy). Instead, we opted to administer docetaxel, the usefulness of which has been reported [9]. The approved docetaxel dose in Japan is 75 mg/m^2^; the dose administered to our patient was 75 mg/m^2^ every 3 weeks.

Since effective adjuvant chemotherapy after curative surgery has not been reported, it was not administered, and the tumor in the left lung recurred 4 months after surgery. Selection of the subsequent chemotherapy drug was challenging. Although tumor regression was slow during the second half of AI therapy, regression was observed; given the lack of effective chemotherapy regimens for this situation, ifosfamide monotherapy was administered. The first course was completed smoothly and the patient was scheduled for discharge; however, her condition deteriorated suddenly, and she died soon thereafter. We believe that a cardiac or other important vessel ruptured during tumor regression; however, we were unable to confirm this hypothesis because an autopsy was not conducted.

Mitus et al. reported that AI therapy was useful for treating breast phyllodes tumors; 1 patient in their study achieved a complete response, and 2 patients achieved a partial response [10]. A complete response was not obtained in our case.

Other possible treatments for a better prognosis included radiation therapy or the administration of another chemotherapy regimen such as a combination of docetaxel and gemcitabine. A dose of 10 g/m^2^ of ifosfamide as a third therapy rather than 8 g/m^2^ in each cycle may also be considered. Furthermore, it is arguable whether the option of surgery is appropriate when the disease has progressed. Moreover, AI therapy has been successful, but further treatment is still controversial.

Given the low frequency with which malignant breast phyllodes tumors occur, distant metastasis, large-scale clinical trials are not feasible. Hence, case reports represent an important means by which to identify effective treatments. Although AI therapy was not curative in our case, it resulted in prominent tumor regression; therefore, this treatment strategy may be considered for use in other cases.

## 4. Conclusion

AI therapy is useful for treating recurrent malignant breast phyllodes tumors.

## Figures and Tables

**Table 1:** Doxorubicin-ifosfamide (AI) therapy regimen.

	Drug	Volume/dose	Infusion time	Timing of administration
*Days 1-2*				
Rp. 1	Saline	500 mL		
	SHC	20 mL	4 h	
Rp. 2	Saline	100 mL		
	PH (day 1 only)	0.75 mg		
	Dexamethasone	9.9 mg	15 min	
Rp. 3	Saline	100 mL		
	Doxorubicin	30 mg/m^2^	2 h	
Rp. 4	Saline	50 mL		
	Mesna	400 mL/m^2^	15 min	
Rp. 5	Saline	500 mL		
	Ifosfamide	2 g/m^2^	4 h	
Rp. 6	Saline	500 mL		
	SHC	20 mL	4 h	
Rp. 7	Saline	50 mL		
	Mesna	400 mL/m^2^	15 min	4 h after Rp. 5 administration
Rp. 8	Saline	500 mL		
	SHC	20 mL	4 h	
Rp. 9	Saline	50 mL		
	Mesna	400 mL/m^2^	15 min	8 h after Rp. 5 administration
*Days 3-5*				
Rp. 1	Saline	500 mL		
	SHC	20 mL	4 h	
Rp. 2	Saline	100 mL		
	Dexamethasone	9.9 mg	15 min	
Rp. 3	Saline	50 mL		
	Mesna	400 mL/m^2^	15 min	
Rp. 4	Saline	500 mL		
	Ifosfamide	2 g/m^2^	4 h	
Rp. 5	Saline	500 mL		
	SHC	20 mL	4 h	
Rp. 6	Saline	50 mL		
	Mesna	400 ml/m^2^	15 min	4 h after Rp. 4 administration
Rp. 7	Saline	500 mL		
	SHC	20 mL	4 h	
Rp. 8	Saline	50 mL		
	Mesna	400 mL/m^2^	15 min	8 h after Rp. 4 administration

Abbreviations: Rp: recipe; SHC: sodium hydrogen carbonate, 8.4%; PH: palonosetron hydrochloride; h: hour; min: minute; AI: doxorubicin-ifosfamide.
